# Comparison of Mechanical Stability of Elastic Titanium, Nickel-Titanium, and Stainless Steel Nails Used in the Fixation of Diaphyseal Long Bone Fractures

**DOI:** 10.3390/ma11112159

**Published:** 2018-11-01

**Authors:** Pei-Yuan Lee, Yen-Nien Chen, Jin-Jia Hu, Chih-Han Chang

**Affiliations:** 1 Department of BioMedical Engineering, National Cheng Kung University, Tainan 70101, Taiwan; b1208b@ms26.hinet.net (P.-Y.L.); changbmencku@gmail.com (C.-H.C.); 2 Department of Orthopedics, Show Chwan Memorial Hospital, Changhua 50008, Taiwan; 3 Medical Device Innovation Center, National Cheng Kung University, Tainan 70101, Taiwan

**Keywords:** titanium, stainless, nitinol, diaphyseal fracture

## Abstract

Elastic nails made of the nickel-titanium shape memory alloy (Nitinol) have been reported to control bone modeling in animal studies. However, the mechanical stability of the Nitinol nail in the fixation of long bone fractures remains unclear. This study compared mechanical stability among nails made of three materials, namely Nitinol, titanium, and stainless steel, in the fixation of long bone fractures. These three materials had identical shapes (arc length: π/2 and radius: 260 mm). A cylindrical sawbone with a 10-mm gap and fixed with two C-shaped elastic nails was used to examine the stability of the nails. A finite element (FE) model was developed based on the sawbone model. The end cap for elastic nails was not used in the sawbone test but was considered based on a constraint equation in FE simulation. The results of stability tests appeared to depend on the presence or absence of the end cap. In the sawbone test, the titanium nail yielded a higher ultimate force against the applied load than did the stainless steel and Nitinol nails before the gap completely closed; the difference in linear stiffness between the nails was nonsignificant. In FE simulation, the titanium nail produced smaller gap shortening than did stainless steel and Nitinol nails without the end cap; the difference in gap shortening between the nails was minor with the end cap. The titanium elastic nail should be a better choice in managing diaphyseal long bone fractures when the end cap is not used. For Nitinol and stainless steel nails, the end cap should be used to stop the nail from dropping out and to stabilize the fractured bone.

## 1. Introduction

The elastic stable intramedullary nail (ESIN) is used for the fixation of diaphyseal long bone fractures in adolescents, children, and small people [1,2,3]. The major advantages of using the ESIN are the minimally invasive nature of the technique, the short operation time, and the preservation of the growth plate. Titanium and stainless steel are commonly used to fabricate elastic nails, and both of these metals result in adequate stabilization of the fractured bone [4,5,6,7]. However, complications, such as bursitis caused by the nail tip, delayed union, and nonunion, commonly occur in patients with body weights of >55 kg or patients aged >13 years [8,9]. Thus, development of an innovative technique or device for treating diaphyseal fractures is still required. In recent years, the nickel-titanium shape memory alloy (SMA) has received increasing attention, because it has been used as a functional intramedullary nail to apply control force during bone remodeling, and as a novel bone plate during osteotomy in animal studies [10,11,12].

Nitinol (Nickel Titanium Naval Ordnance Laboratory) is a type of SMA and has been widely used in many implant types, such as vascular stents and memory staples [13,14], as well as in medical instruments, such as the Nitinol rod used for treating scoliosis, tissue ablation devices, and endoscopes [15,16,17]. Nitinol is the most widely used SMA in medical applications because of its excellent biocompatibility, mechanical properties, and anticorrosion ability [18,19,20]. Furthermore, according to the American Society for Testing and Materials (ASTM F2063), the phase transformation temperature of Nitinol is close to the human body temperature. Nitinol is also widely used because of its unique shape memory effect and superelastic behavior. These two phenomena are attributed to the alloy’s phase changes (Martensite phase ↹ Austin phase). The difference between the shape memory effect and superelastic behavior is the starting temperature of phase transformation, which is dependent upon the proportion of nickel and titanium, as well as the thermomechanical parameters used during manufacture. 

The Nitinol nail has been demonstrated to increase bone density and promote bone healing in animal models [10,11]. Furthermore, in human beings, Nitinol staples are used in the fixation of bone fractures for bones that bear relatively little weight, such as the carpal bones and phalanges, and these applications are associated with favorable outcomes [13,21]. However, the Nitinol nail is rarely used to fix long bone fractures of the lower limb, and the mechanical stability of the Nitinol nail in the fixation of long bone fractures has not been studied. The difference in mechanical stability between the currently used titanium and stainless steel elastic nails and the Nitinol nail in the fixation of long bone fractures, particularly in the lower limbs, must be clarified before clinical application. A previous finite element (FE) study reported the importance of the prebending degree of titanium nails in the fixation of long bone fractures [22]. A study reported that the effect of the end cap is crucial to mechanical stability when the prebending degree is not adequate to apply sufficient frictional force to stop the nail from sliding [23]. However, only the titanium nail was examined in these studies; the Nitinol and stainless steel nails were not considered. Therefore, in the present study, the mechanical stability of the Nitinol nail was compared with that of the currently used titanium and stainless steel nails in the fixation of diaphyseal long bone fractures by using the sawbone test and FE simulation. FE simulation is a numerical method used to solve complex nonlinear problems, and it has been used in many types of mechanical studies [24,25]. The present study hypothesized that there is no difference in mechanical stability between Nitinol, titanium, and stainless steel nails in the fixation of diaphyseal long bone fractures.

## 2. Materials and Methods

To investigate the effect of various nail materials, namely titanium, stainless steel, and Nitinol, on the stability of the fractured long bone fixed with two C-shaped nails, an experimental test and FE simulation were used.

### 2.1. Experimental Test

**Figure 1:**
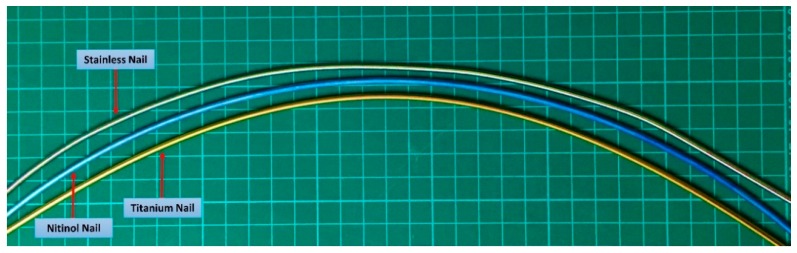
C-shaped nails used in this study.

**Figure 2:**
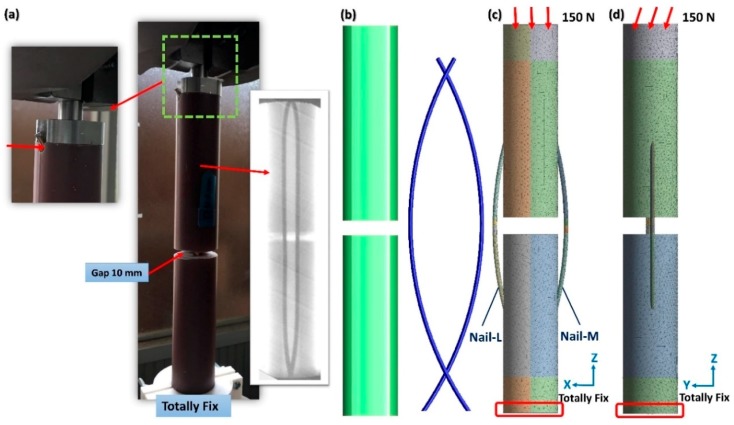
Sawbone model and experimental setup (**a**); The three-dimensional models of the nail and bone (**b**); Boundary conditions of axial compression (**c**) and bending (**d**).

A cylindrical sawbone (Pacific Research Laboratories, Vashon, Washington, WA, USA) with a length of 260 mm, an outer diameter of 35 mm, and a wall thickness of 11 mm was used, and a 10-mm transverse gap was created at the middle of the sawbone. Subsequently, two prebent, C-shaped elastic nails were used to fix the fractured sawbone. The outer diameters of the nails was 3 mm [26]; this size is widely used in clinical settings. The nails had arc lengths of π/2 and radiuses of 260 mm (Figure 1). Holes were created using a 4-mm drill bit at the distal border to insert the nails, and the holes were located 45° to the long axis of the sawbone. A commercial titanium elastic nail (DePuy Synthes, Oberdorf, Switzerland) was used, and the stainless steel nail (316 L) and Nitinol nail (weight percentage of nickel and titanium: 56.1% and 43.9%, respectively) were manufactured by Nitinol Innovative Technology Co., Ltd. (Kaohsiung Taiwan). The curves of the nails made of different materials were set using a mold that was custom made by Nitinol Innovative Technology Co., Ltd. The commercial end cap that is typically used to prevent the nail from dropping out was not used in this experiment; thus, the nail end could be dropped out of the nail under loading (Figure 2a). A total of 18 samples, divided into three groups (6 samples in each group), were used in this experiment. 

A material property test system (MTS 858; MTS Systems Corporation, Eden Prairie, MN, USA) was used to apply axial compression force to the fractured sawbone fixed with two C-shaped elastic nails (Figure 2a). The distal end of the sawbone was completely fixed. The loading rate was set to 1 mm/min, and loading was applied until the gap closed fully. The environmental temperature was set to 24°C by using an air conditioning system. 

### 2.2. FE Simulation

Based on the present sawbone nail model, a solid model was developed using SolidWorks 2014 (Dassault Systemes SolidWorks Corp., Waltham, MA, USA) to examine the internal responses of the nail and bone under loading. The outer diameter and prebending degrees of the nail were the same as those used in the experimental setting (Figure 2b). The nail close to the coordinate origin on the X axis was defined as Nail-M, and the other nail was defined as Nail-L. The three-dimensional (3D) model was then imported into ANSYS Workbench V17 (Swanson Analysis Systems, Inc., Houston, PA, USA) to generate the FE model. Ten-node quadratic tetrahedron elements (solid 187) were used to mesh the whole model. Compared with the linear element, quadratic elements can simulate the deformation of a bone and nail more precisely, and tetrahedron elements are the most powerful elements for meshing the complex geometry of a bone and nail. The element edges of the bone and nail were set to 2 and 1 mm, respectively, by using the command “sizing” in Workbench. The same command was used to set the element edge of inner surfaces that were in contact with the nail to 1.5 mm. A total of 151,554 nodes and 89,396 elements were used in the simulation. The contact behaviors between the nail and the canal, between the bone fragments (at the fracture site), and between the nails were all set to frictional surface-to-surface contact behaviors (target 174 and contact 170 in ANSYS). The frictional coefficients of the bone-to-bone, nail-to-bone, and nail-to-nail were set to 0.45, 0.3, and 0.2, respectively [27,28]. The use of the end cap was considered; however, 3D modeling of the end cap was not conducted. The effect of using the end cap to bond the distal surfaces of nails to surrounding bones was simulated using a constraint equation. The distal end of the nail was set to free if the end cap was not used.

**Figure 3:**
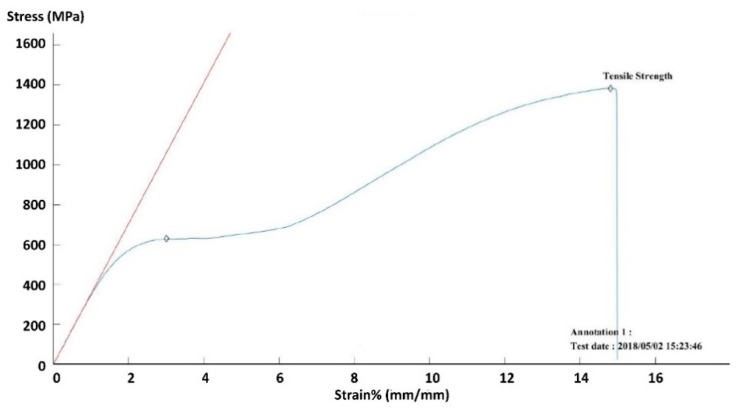
Relationship between the stress and strain of Nitinol rod used in this study.

The elastic modulus and Poisson’s ratio of the bone were 12 GPa and 0.3 (according to the manufacturer), respectively. Titanium and stainless steel were set as plastic behavior, and the bilinear hardening effect was then adopted for these two metals. Material parameters were used from the engineering database in ANSYS Workbench (Table 1). The superelastic material property of Nitinol was used in this simulation, and material parameters were defined by the curve fitting of the stress-strain relationship (Figure 3) observed during the tensile test of the Nitinol rod at room temperature (24 °C). 

Two types of loading conditions based on our previous study were used to examine the stability of the fractured bone fixed with nails fabricated from different materials [22]. To simulate the loading conditions of the lower limb in gait, a force of 150 N was applied to the superior surface of the bone model in the following two directions: vertical (axial compression) and 10° to the vertical line (bending) (Figure 2c,d). The distal end was completely fixed. The nails and bone exhibited initial partial volume interference; thus, volume interference was excluded by the program during the first step, and after the first step, the nails were constrained inside the intramedullary canal. The process of inserting the nails into the canal was simulated in this step, and a constraint force was developed from the canal and applied on the nails. The aforementioned loading of 150 N was applied on the bone in the subsequent loading step.

### 2.3. Incidence and Data Analysis

**Figure 4:**
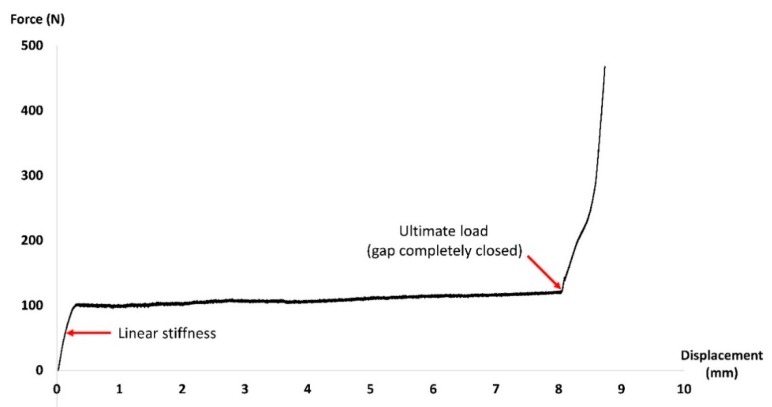
Relationship between the displacement and applied force of the sawbone with nails.

In the experimental compression test, the loading with the gap completely closed was recorded as “ultimate load,” and the relationship between force and displacement at the initial linear stage was referred to as “linear stiffness” for comparison (Figure 4). The value of linear stiffness was calculated using linear regression. One-way ANOVA was used to test for significance between different nails and a P value of less than 0.05 was used. In FE simulation, gap shortening under compression was used as an index for stability. Under bending, the compression (shortened) side of the gap was used as an index. 

## 3. Results

### 3.1. Experimental Test

**Figure 5:**
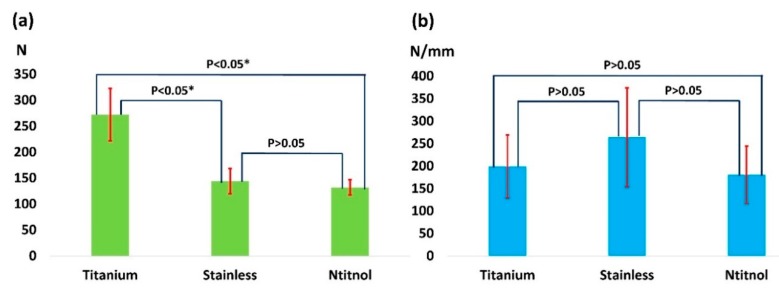
Ultimate load (**a**) and linear stiffness (**b**) of nails.

Experimental results indicated that compared with stainless steel and Nitinol nails, the titanium nail yielded a significantly higher ultimate load before the gap completely closed (P < 0.05); the difference in the ultimate load between stainless steel and Nitinol nails was not significant (Figure 5). Furthermore, no significant difference in linear stiffness was observed among titanium, stainless steel, and Nitinol nails. The average ultimate loads yielded by titanium, stainless steel, and Nitinol nails were 272 (SD: 50), 144 (SD: 24), and 111 (SD: 15) N, respectively. Additionally, the average linear stiffness values yielded by the titanium, stainless steel, and Nitinol nails were 199 (SD: 70), 264 (SD: 110), and 180 (SD: 64) N/mm, respectively.

### 3.2. FE Simulation

**Figure 6:**
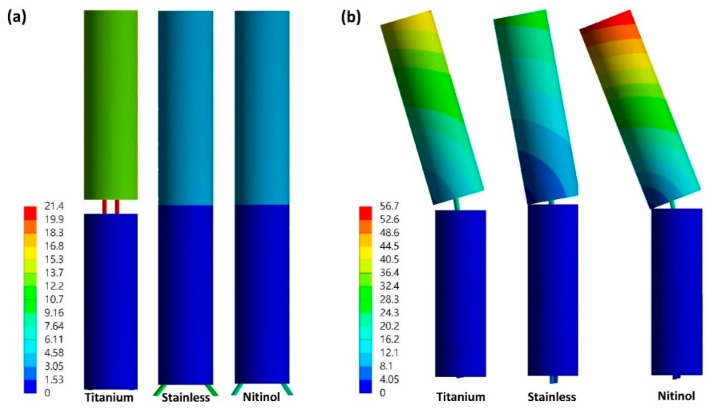
Total deformation (mm) of the fractured bone with nails and without end cap under compression (**a**) and bending (**b**).

**Figure 7:**
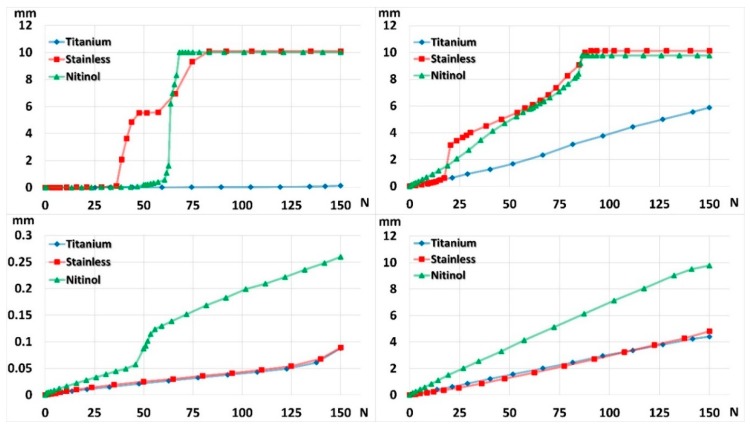
Gap shortening with (**bottom row**) and without (**top row**) end cap in compression (**left column**) and bending (**right column**).

FE simulation results indicated that the titanium nail could sustain the applied loading (150 N) without gap closing, either under axial compression or bending (Figure 6a,b), in the absence of the end cap. By contrast, stainless steel and Nitinol nails could not maintain the gap when 150 N of loading was fully applied in the absence of the endcap, and the gap closed completely during the process of load application. The gap fully closed at 82 and 68 N under axial compression, and at 90 and 86 N under bending for stainless steel and Nitinol nails, respectively (Figure 7). When the end cap was used, gap deformation was larger for the Nitinol nail than for titanium and stainless steel nails; the difference in gap deformation was not minor between titanium and stainless steel nails (Figure 7). The end cap could stop the gap from closing. In addition, the titanium nail developed a higher contact force between the nail and the bone than did stainless and Nitinol nails after the insertion of the nail (Table 2). The difference in the contact force between Nitinol and stainless steel nails was very minor.

## 4. Discussion

In this study, the mechanical stability of the currently used titanium and stainless steel nails was compared with that of the Nitinol nail in the fixation of diaphyseal long bone fractures by using the sawbone test and FE simulation. The results of the sawbone test and FE simulation both indicated that the titanium nail provided the highest stability for the bone fracture, particularly when the end cap was not used. The Nitinol nail demonstrated the lowest stability. Although the Nitinol nail has facilitated a favorable outcome for bone union in animal studies [10,11], it provided lower mechanical stability than did titanium and stainless steel nails in the fixation of long bone fractures in the present study.

The titanium nail resulted in a higher ultimate load (sawbone test) and stability (FE simulation) than did stainless steel and Nitinol nails, because the yield stress of titanium (800 MPa) is considerably higher than that of stainless steel (210 MPa). Although the elastic modulus of titanium in the linear phase is only half of that of stainless steel, the linear elastic zone (before the yield stress) of titanium is much wider than that of stainless steel. Compared with the stainless steel nail, the titanium nail developed a higher contact force, thereby generating a higher frictional force against the applied load. The superelastic property of Nitinol could supply a consistent force when phase transformation was triggered, and the force did not increase with strain. Although the linear zone (before entering the martensite phase; 600 MPa) of Nitinol is slightly smaller than that of titanium, the elastic modulus of Nitinol is considerably lower than that of titanium. Compared with the titanium nail, the Nitinol nail developed a lower contact force between the nail and canal; consequently the Nitinol nail’s ultimate force was also lower.

Titanium and stainless steel are commonly used metals for preparing ESINs. Nails composed of stainless steel and titanium may exhibit different responses under loading. Whether the titanium nail provides higher stability than the stainless steel nail remains controversial [29,30]. In the present study, the titanium nail resulted in lower gap deformation than did the stainless steel nail, which is consistent with the result reported by Perez [7,29]. Furthermore, compared with the stainless steel nail, the titanium nail provided a higher contact force between the nail and canal, resulting in a higher frictional force against the applied load. The prebending degree, which was not considered in Perez’s simulation, was considered in the present simulation. The stainless steel nail provided higher structural stiffness than did the titanium nail in Kaiser’s study [30]. Kaiser’s result is in accordance with the results on linear stiffness in the present study. The average value of linear stiffness in this study was higher for the stainless steel nail than for the titanium nail, but the variance for each nail type was large. The large variance (70 and 110 N/mm in titanium and stainless steel, respectively) may explain why stainless steel nails have yielded higher stability in some studies, whereas other studies have reported that titanium nails yielded higher stability. Therefore, the ultimate load, which was first demonstrated in the present study, is another measure for determining the effect of nail properties on mechanical stability.

In previous FE simulation studies, the end cap was reported to be crucial to the stability of the titanium nail when the contact force between the nail and canal was not adequate to stop the nail from sliding against the inner aspect of the canal, particularly in nails with low prebending degrees [22,23]. In this study, the contact force developed by the titanium nail, which had an arc length of π/2 and a radius of 260 mm, was adequate to generate sufficient frictional force against the applied loading of 150 N and maintain the gap without the end cap. However, stainless steel and Nitinol nails could not develop sufficient contact and frictional forces to resist the applied load without the end cap, resulting in the closure of the gap and the dropping out of the nail. When the end cap was used, it provided adequate support to the nail end and prevented the nail from dropping out. Thus, the use of the end cap is recommended if the stainless steel or Nitinol nail is used for the management of diaphyseal long bone fractures. 

Although the Nitinol nail demonstrated relatively low stability in this study, it remains a potential biomaterial for many medical devices because of its unique shape memory effect and superelastic material properties. Furthermore, compared with the elastic moduli of titanium (110 GPa) and stainless steel (210 GPa), the elastic modulus of Nitinol (30–70 GPa) is much closer to that of the cortical bone. Hence, the stress shielding effect caused by a material with a higher elastic modulus than that of the bone can be decreased by reducing the elastic moduli of implants. The shape memory effect enables the implant to be delivered into the bone through a small tunnel, and then recovery to the original complex shape for supporting, such as kyphoplasty [31,32]. In addition, the shape memory effect can be used to generate and apply a compressive force to the fracture site of non-weight-bearing bones, such as metatarsal and carpal bones [33,34]. Absolute stiffness is not necessary for non-weight-bearing bones, and force stimulation can contribute to bone healing [35].

The elastic modulus of Nitinol used in this study was only approximately 30 GPa, which was lower than that reported in the literatures [36,37,38]. In Taiwan, the available source of the Nitinol alloy is rarer than those of the commonly used titanium and stainless steel alloys. In the future, the stability of the Nitinol nail may be increased if the elastic modulus of the Nitinol alloy is increased. 

This study involved some limitations that should be addressed. First, the simulation results are approximate solutions, and some small errors likely occurred during the transformation of the complex geometry into simple elements. Second, the FE bone model was simplified as a cylinder for comparison with the sawbone. The real contact between the nail and canal is more complex than that in the present model. Third, the effect of temperature on the material properties of Nitinol was not considered in the FE simulation. Fourth, the solid model of the end cap was not modeled, and the contribution of the end cap was replaced by a constraint equation in the simulation. Fifth, the difference of frictional coefficient between the materials was not considered in the simulation. Sixth, the Nitinol with a higher elastic modulus is unavailable, hence the stability may be underestimated. We have substituted the elastic modulus of the Nitinol with a higher value in the simulation and it indeed increased the stability.

## 5. Conclusions

Nitinol has received substantial attention in recent years, particularly for its applications in orthopedic implants. Thus, the differences in mechanical behavior between Nitinol and conventional metals should be clarified before this material is further applied clinically, particularly in weight-bearing limbs. The present study is the first to demonstrate the mechanical stability of titanium, stainless steel, and Nitinol nails in the fixation of diaphyseal long bone fractures. According to the results of this study, the C-shaped titanium elastic nail should be the top choice for the management of diaphyseal long bone fractures when the end cap is not used. For Nitinol and stainless steel nails, use of the end cap is suggested to prevent the nail from dropping out and to stabilize the fractured bone. Additionally, loading with the gap completely closed (the ultimate load proposed in this study) is a key index—along with structural stiffness and gap deformation—for judging the stability of elastic stable intramedullary nails fabricated from various materials. 

## Figures and Tables

**Table 1:** Material properties of titanium and stainless steel used in this study.

Material	Elastic Modulus (MPa)	Poisson Ratio	Yield Strength (MPa)	Tangent Modulus (MPa)
Titanium	110,000	0.3	800	1250
Stainless	200,000	0.3	210	1800

**Table 2:** Initial contact force (N) between the canal and nails fabricated from various metals.

Nail	Titanium	Stainless	Nitinol
Bone shaft			
Nail-M	107.7	34.2	37.2
Nail-L	108.5	31.5	36.7
Inserting hole			
Nail-M	141	51	53
Nail-L	143.4	45.7	49.2
